# Metabolomics profile in acute respiratory distress syndrome by nuclear magnetic resonance spectroscopy in patients with community-acquired pneumonia

**DOI:** 10.1186/s12931-022-02075-w

**Published:** 2022-06-27

**Authors:** Yongqin Yan, Jianuo Chen, Qian Liang, Hong Zheng, Yiru Ye, Wengang Nan, Xi Zhang, Hongchang Gao, Yuping Li

**Affiliations:** 1 Department of Respiratory and Critical Care Medicine, The First Affiliated Hospital of Wenzhou Medical University, Nanbaixiang Street, Wenzhou, 325000 China; 2 Institute of Metabonomics & Medical NMR, School of Pharmaceutical Sciences, Wenzhou Medical University, Wenzhou, China

**Keywords:** Acute respiratory distress syndrome, Community-acquired pneumonia, Metabolomics, Nuclear magnetic resonance

## Abstract

**Background:**

Acute respiratory distress syndrome (ARDS) is a challenging clinical problem. Discovering the potential metabolic alterations underlying the ARDS is important to identify novel therapeutic target and improve the prognosis. Serum and urine metabolites can reflect systemic and local changes and could help understanding metabolic characterization of community-acquired pneumonia (CAP) with ARDS.

**Methods:**

Clinical data of patients with suspected CAP at the First Affiliated Hospital of Wenzhou Medical University were collected from May 2020 to February 2021. Consecutive patients with CAP were enrolled and divided into two groups: CAP with and without ARDS groups. ^1^H nuclear magnetic resonance-based metabolomics analyses of serum and urine samples were performed before and after treatment in CAP with ARDS (n = 43) and CAP without ARDS (n = 45) groups. Differences metabolites were identifed in CAP with ARDS. Furthermore, the receiver operating characteristic (ROC) curve was utilized to identify panels of significant metabolites for evaluating therapeutic effects on CAP with ARDS. The correlation heatmap was analyzed to further display the relationship between metabolites and clinical characteristics.

**Results:**

A total of 20 and 42 metabolites were identified in the serum and urine samples, respectively. Serum metabolic changes were mainly involved in energy, lipid, and amino acid metabolisms, while urine metabolic changes were mainly involved in energy metabolism. Elevated levels of serum 3-hydroxybutyrate, lactate, acetone, acetoacetate, and decreased levels of serum leucine, choline, and urine creatine and creatinine were detected in CAP with ARDS relative to CAP without ARDS. Serum metabolites 3-hydroxybutyrate, acetone, acetoacetate, citrate, choline and urine metabolite 1-methylnicotinamide were identified as a potential biomarkers for assessing therapeutic effects on CAP with ARDS, and with AUCs of 0.866 and 0.795, respectively. Moreover, the ROC curve analysis revealed that combined characteristic serum and urine metabolites exhibited a better classification system for assessing therapeutic effects on CAP with ARDS, with a AUC value of 0.952. In addition, differential metabolites strongly correlated with clinical parameters in patients with CAP with ARDS.

**Conclusions:**

Serum- and urine-based metabolomics analyses identified characteristic metabolic alterations in CAP with ARDS and might provide promising circulatory markers for evaluating therapeutic effects on CAP with ARDS.

**Supplementary Information:**

The online version contains supplementary material available at 10.1186/s12931-022-02075-w.

## Background

Acute respiratory distress syndrome (ARDS) is a clinical syndrome characterized by acute inflammation, pulmonary vascular hyperpermeability, and pulmonary edema secondary to direct or indirect lung injury, including sepsis, pneumonia, trauma, aspiration pneumonia, pancreatitis, and other critical clinical conditions[1–3]. Community-acquired pneumonia (CAP) is a common cause of ARDS[4]. CAP-induced ARDS remains a challenging clinical problem that causes high morbidity, mortality, and financial burden worldwide[5, 6]. CAP has acute onset and manifests as dyspnea and lung infiltration, similar to ARDS. To date, the characteristic metabolic changes of CAP with ARDS are unknown. Finding different metabolites in CAP with and without ARDS is important to identify novel therapeutic target and improve the prognosis of the disease.

Metabolomics is an emerging approach for identifying and quantifying metabolites in biofluids, cells, and tissues. It can detect subtle alterations in biological pathways, thus providing insight into mechanisms underlying both physiological and pathological processes and correlating them with clinical outcomes[7, 8]. Decreased serum glucose, alanine, glutamine, methylhistidine and fatty acids concentrations, and elevated serum phenylalanine and methylguanidine concentrations help identify ARDS in patients with H1N1 influenza A virus pneumonia using nuclear magnetic resonance (NMR) imaging[9]. Twenty-three candidate metabolic biomarkers were identified in ARDS caused by H1N1 influenza A virus, including lower levels of lysophospholipids, tryptophan and higher level of bile acids metabolites, suggesting that metabolomics can demonstrate the specificity of major alterations in ARDS caused by H1N1 influenza A virus infection[2]. Methylpent-2-enal, 2,4-octadiene 1-chloroheptane, and nonanal in exhaled breath may reflect metabolic characterization of coronavirus disease patients with ARDS using metabolomics[10]. These data suggest that metabolomics can reflect systemic and local changes and serve as major alterations for CAP-induced ARDS.

The aim of the present study was to identify panels of significant metabolites for reflecting characteristic metabolic alterations and evaluating therapeutic effects on CAP with ARDS. We performed ^1^H NMR-based metabolomics of serum and urine samples from patients with CAP.

## Methods

### Study design

**Fig. 1:**
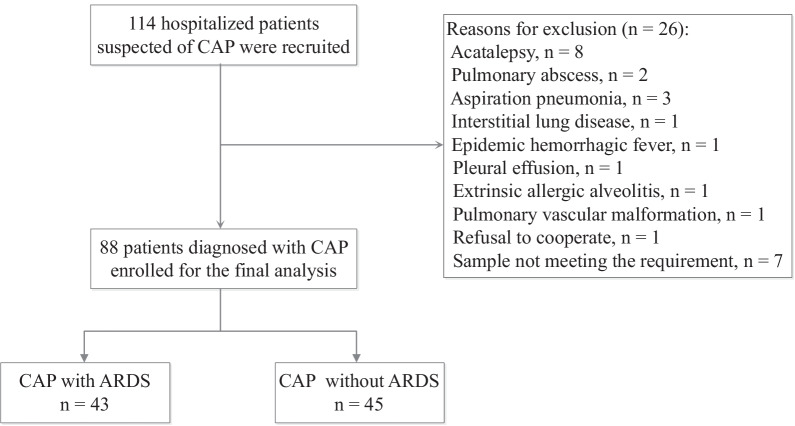
Flowchart of study population enrolment. *CAP* community-acquired pneumonia, *ARDS* acute respiratory distress syndrome

This prospective observational study was conducted at the First Affiliated Hospital of Wenzhou Medical University, China, from May 2020 to February 2021. We consecutively enrolled 114 hospitalized patients suspected with CAP, 26 of whom were excluded. They were divided into two groups: CAP with and without ARDS groups. ARDS was diagnosed according to the Berlin definition within 24 h of hospital admission (Fig. 1)[5].

CAP was diagnosed according to the 2016 CAP guidelines from China[11]: A. onset in community; B. clinical manifestations of pneumonia: (1) new onset of cough or expectoration or aggravation of existing symptoms of respiratory tract diseases, with or without purulent sputum, chest pain, dyspnea, or hemoptysis, (2) fever, (3) signs of pulmonary consolidation and/or moist rales, and (4) peripheral white blood cell (WBC) count > 10 × 10^9^/L or < 4 × 10^9^/L, with or without left shift; and C. chest radiograph showing new patchy infiltrates, lobar or segmental consolidation, ground-glass opacities, or interstitial changes, with or without pleural effusion. CAP was diagnosed when a patient satisfied criteria A and C and any one condition of criterion B and was ruled out for tuberculosis, pulmonary tumors, non-infectious interstitial lung disease, pulmonary edema, atelectasis, pulmonary embolism, pulmonary eosinophilia, and pulmonary vasculitis. Moreover, patients who refused to cooperate were excluded.

All patients received inpatient treatment according to CAP guidelines. We collected serum and urine samples before and after treatment, when the condition had improved from baseline. Clinical data, including sex, age, underlying diseases, laboratory findings, and treatment were recorded, and missing clinical data were filled by regression estimation. The study protocol was approved by the Ethics Committee of the First Affiliated Hospital of Wenzhou Medical University (No. 2020–111). The study was conducted following the tenets of the Declaration of Helsinki (as revised in 2013). Informed consent was obtained for experimentation with human subjects.

### Sample collection and storage

All participants were in an overnight fasting state, and in the morning, peripheral venous blood was obtained in a 5 mL BD Vacutainer® tube containing the chelating agent ethylenediamine tetraacetic acid. Serum samples were separated by centrifugation at 3,000 × g for 15 min at 4 °C and stored at − 80 °C until metabolomics was analyzed using NMR spectroscopy. Similarly, fasting midstream clear urine samples were obtained during a standard routine clinical procedure. Urine samples were centrifuged at 10,000 × g for 10 min at 4 °C and stored at − 80 °C until metabolomics was analyzed using NMR spectroscopy.

### Metabolomic analysis using NMR spectroscopy

We thawed 200 µL serum samples at 4 °C and diluted them with 250 µL phosphate-buffered saline and 50 µL deuterium oxide (D_2_O). Simultaneously, 200 µL urine samples were thawed at 4 °C diluted with 50 µL of D_2_O containing 0.2 mM sodium salt of 3-(trimethylsilyl) propionic-2,2,3,3,d4 (TSP) acid and 250 µL phosphate-buffered saline. After vortexing, samples were centrifuged at 12,000 rpm for 10 min. Subsequently, all supernatants were transferred into 5 mm NMR tubes and analyzed.

### NMR-based metabolomics analysis

^1^H NMR-based metabolomics analysis was performed using a 600-MHz Bruker Avance III NMR spectrometer equipped with a 5-mm TXI probe (Bruker BioSpin, Rheinstetten, Germany) at 25 °C. A standard CPMG pulse sequence with a fixed receiver-gain value was used to acquire the NMR spectra to minimize the line-broadening effects of the residual water signal. The brief duration of the CPMG sequence made the effect on the quantitative nature of the experiment negligible. The main acquisition parameters for serum were set as follows: data points, 160 K; relaxation delay, 4 s; spectral width, 12,335.5 Hz; and acquisition time, 2.66 s/scan. In addition, the ^1^H NMR spectra of urine were recorded using the NOESY gradient pulse sequence with 256 transients. A sweep width of 10,822.5 Hz was acquired with 256 data points at an acquisition time of 2.66 s.

All NMR spectra were manually corrected for phase and baseline using Topspin 3.0 software (Bruker BioSpin, Rheinstetten, Germany). Urine spectra were referenced to the TSP acid peak at 0.00 ppm, while serum spectra were referenced to the lactate signal at 1.33 ppm. Metabolites in the NMR spectrum were assigned using Chenomx NMR suite 7.0 (Chenomx Inc., Edmonton, Canada). All spectra were processed using MATLAB R2012a (MathWorks Inc.). The spectra were phase-adjusted, baseline-corrected, and integrated using the “icoshift” procedure and overall normalized. The spectra from 0.6 to 9.0 ppm excluding the residual water region (4.6 to 5.1 ppm) were subdivided and integrated to binning data with sizes of 0.01 and 0.0015 ppm for multivariate and quantitative analyses, respectively.

For the multivariate analysis, the orthogonal partial least-squares discriminant analysis (OPLS-DA) was conducted using Pareto-scaled NMR data in SIMCA 12.0 software (Umetrics, Umeå, Sweden). Moreover, a permutation test (20 cycles) was conducted to evaluate the performance of OPLS-DA, in which R2 and Q2 were calculated as the model’s goodness of fit and predictive capability, respectively. The OPLS-DA score plot exhibited differences in metabolic patterns between groups. For the quantitative analysis, relative concentrations of identified metabolites were quantified using their peak areas.

Key metabolites were screened based on a variable importance in projection (VIP) > 3 and false discovery rate (FDR) < 0.05 in serum and VIP > 1 and FDR < 0.05 in urine. The receiver operating characteristic (ROC) curve analysis was performed to evaluate the diagnostic efficacy of significant metabolites using MedCalc 19.0 software. Metabolic pathways were produced manually using Adobe Photoshop CS6 (Adobe Inc., San Jose, CA) at the base of the KEGG database. The correlation heatmap was produced using the R programming language (4.1.3).

### Statistical analysis

Among data, qualitative variables are expressed as frequency (percentage), and continuous variables are expressed as mean (standard deviation) or median (interquartile range). The independent-samples *t*-test (for normally distributed data) and Mann–Whitney U test (for non-normally distributed data) were performed to compare CAP with and without ARDS groups. Differences between patients with ARDS before and after treatment were analyzed using the paired samples *t*-test (for normally distributed data) and Wilcoxon signed-rank test (for non-normally distributed data). All statistical analyses were performed using SPSS (IBM SPSS Statistics 26), and FDR < 0.05 was considered to be statistically significant.

## Results

### Demographic and clinical characteristics of participants

We enrolled 88 patients, including 43 and 45 patients in CAP with and without ARDS groups, respectively. Table 1 shows the demographic and clinical characteristics. Additional file 1: Table S1 shows the dynamic clinical parameters before and after treatment. After treatment, the CAP with ARDS group showed significantly reduced WBC count, absolute neutrophil count, hemoglobin level, aspartate aminotransferase level, total bilirubin level, blood urea nitrogen level, serum creatinine level, glucose level, creatine kinase level, lactate dehydrogenase level, interleukin-6 level, interleukin-10 level, interferon-γ level, C-reactive protein (CRP) level, procalcitonin level, erythrocyte sedimentation rate, and ferritin level and significantly elevated absolute lymphocyte count, platelet count, CD4 + T cells, CD8 + T cells, and total protein, albumin, total cholesterol, and low-density lipoprotein levels.

**Table 1:** Clinical and demographic characteristics of patients with community-acquired pneumonia with and without acute respiratory distress syndrome

Variables	CAP with ARDS (n = 43)	CAP without ARDS (n = 45)
Male sex	30 (69.8)	30 (66.7)
Age (years)	63.9 ± 12.4	60.8 ± 10.8
Smokers	19 (44.2)	19 (42.6)
Underlying diseases		
Cardiovascular disease	25 (58.1)	20 (44.4)
Diabetes mellitus	20 (46.5)	12 (26.7)
Cerebrovascular disease	3 (7.0)	1 (2.2)
Chronic kidney disease	2 (4.7)	6 (13.3)
PSI score	20 (10–30)	10 (0–20)
APACHE II score	12 (10–18)	6 (3.0–9.3)
Laboratory results		
WBC (× 10^9/L)	10.2 (5.9–13.1)	7.6 (5.5–9.5)
LDH (µ/L)	373 (259.6–502.0)	260 (221.2–354.5)
IL-6 (µg/L)	208.3 (97.4–1783.9)	46.7 (15.5–127.4)
IL-10 (µg/L)	8.3 (5.2–29.1)	3.3 (2.1–6.1)
LAC (mmol/L)	2.4 (1.8–4.0)	1.8 (1.3–2.7)
CRP (mg/dL)	183 (146–215)	94.7 (57.3–151.5)
PCT (ng/mL)	5.7 (0.9–18.3)	0.2 (0.1–0.7)
Oxygen therapy and respiratory support		
Nasal cannula	18 (41.8)	45 (100.0)
Mask and/or HFNO	5 (11.6)	0 (0.0)
Noninvasive ventilation	8 (18.6)	0 (0.0)
Invasive mechanical ventilation	12 (27.9)	0 (0.0)
Outcome		
Death	7 (16.3)	0 (0.0)
Survival	36 (83.7)	45 (1.0)

*CAP* community-acquired pneumonia, *ARDS* acute respiratory distress syndrome, *PSI* pneumonia severity index, *APACHE II* acute physiology and chronic health evaluation II scores, *WBC* white blood cell, *LDH* lactate dehydrogenase, *IL* interleukin, *LAC* lactic acid, *CRP* c-reactive protein; *PCT* procalcitonin, *HFNO* high-flow nasal oxygen

### Differences metabolites between CAP with and without ARDS groups in serum and urine and dynamic changes in CAP with ARDS group after treatment

**Fig. 2:**
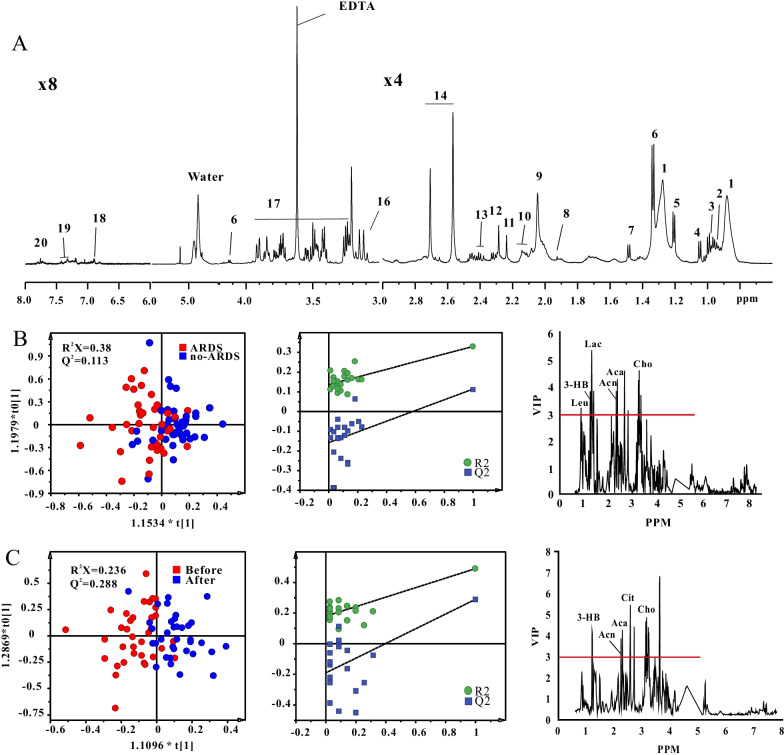
Metabolomics analysis of serum samples. **A** Typical ^1^H nuclear magnetic resonance spectrum. 1. low-density lipoprotein/very-low-density lipoprotein; 2. leucine; 3. isoleucine; 4. valine; 5. 3-hydroxybutyric acid; 6. lactate; 7. alanine; 8. acetate; 9. lipid; 10. glutamine; 11. acetone; 12. acetoacetate; 13. pyruvate; 14. citrate; 15 Creatine. creatine; 16. choline; 17. glucose; 18. tyrosine; 19. phenylalanine; 20. methylhistidine. Score scatter plots of orthogonal partial least-squares discriminant analysis from serum samples and variable importance in projection shows the difference between **B** community-acquired pneumonia with and without acquired respiratory distress syndrome groups; and **C** before and after treatment in the community-acquired pneumonia with acquired respiratory distress syndrome group. *Leu* leucine, *3-HB* 3-hydroxybutyrate, *Lac* lactate, *Acn* acetone, *Cit* citrate, *Cho* choline, *Aca* acetoacetate

**Fig. 3:**
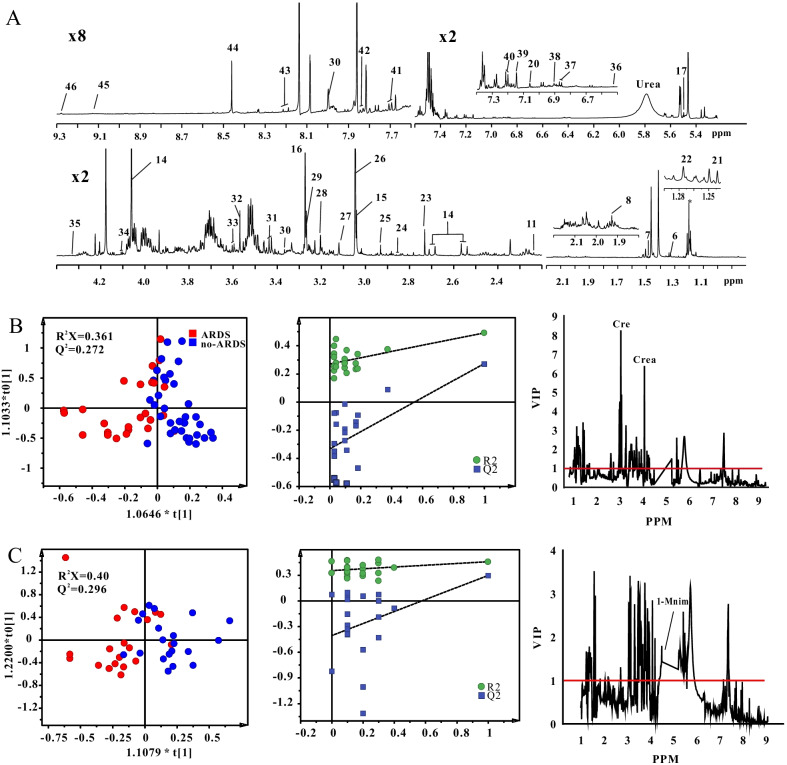
Metabolomics analysis of urine samples. **A** Typical ^1^H nuclear magnetic resonance spectrum. 6. lactate; 7. alanine; 8. acetate; 9. lipid; 10. glutamine; 11. acetone; 12. acetoacetate; 13. pyruvate; 14. citrate; 15. creatine; 16. choline; 17. glucose; 18. tyrosine; 19. phenylalanine; 20. methylhistidine; 21. fructose; 22. 3-hydroxyisovalerate; 23. dimethylamine; 24. asparagine; 25. N, N-dimethylglycine; 26. creatinine; 27. malonate; 28. carnitine; 29. betaine; 30. theophylline; 31. taurine; 32. glycine; 33. threonine; 34. fructose; 35. tartrate; 36. Fumarate; 37. tyrosine; 38. vanillate; 39. histidine; 40. tryptamine; 41. 3-indoxyl sulfate; 42. hippurate; 43. hypoxanthine; 44. formate; 45. trigonelline; 46. 1-methylnicotinamide. Score scatter plots of orthogonal partial least-squares discriminant analysis and variable importance in projection shows a difference between **B** community-acquired pneumonia with and without acquired respiratory distress syndrome groups; and **C** before and after treatment in the community-acquired pneumonia with acquired respiratory distress syndrome group. *Cre* creatine, *Crea* creatinine, *1-Mnim* 1-methylnicotinamide

A total of 20 metabolite signals were identified from the NMR-based serum metabolome (Fig. 2A), including lipid (choline, lipid, and low-density lipoprotein/very-low-density lipoprotein), energy (glucose, citrate, lactate, pyruvate, acetate, acetone, acetoacetate, creatine, and 3-hydroxybutyrate), and amino acid (leucine, isoleucine, phenylalanine, valine, alanine, glutamine, methylhistidine, and tyrosine) metabolisms. In addition, a total of 42 metabolite signals were identified from ^1^H NMR-based urine spectra (Fig. 3A), mainly including lipid (acetate and choline), energy (fructose, citrate, lactate, pyruvate, acetone, creatine, creatinine, etc.), and amino acid (phenylalanine, taurine, alanine, glutamine, methylhistidine, 3-indoxyl sulfate, tryptamine, tyrosine, etc.) metabolisms.

The metabolites were quantitatively and statistically analyzed. As for energy metabolism, compared to the CAP without ARDS group, the CAP with ARDS group had higher levels of serum lactate, creatine, pyruvate, 3-hydroxybutyric acid, acetone, and acetoacetate and lower levels of urine creatinine, creatine, and 1-methylnicotinamide. As for amino acid metabolism, compared to the CAP without ARDS group, the CAP with ARDS group had lower levels of serum alanine, leucine, isoleucine, and glutamine and higher levels of serum phenylalanine. As for lipid metabolism, compared to the CAP without ARDS group, the CAP with ARDS group had lower levels of serum choline (Additional file 1: Table S2).

After treatment in the CAP with ARDS group, energy metabolism changed: Serum lactate, pyruvate, acetoacetate, acetone, and 3-hydroxybutyric acid levels reduced, whereas serum citrate and urine 1-methylnicotinamide levels elevated. Similarly, amino acid metabolism changed: Serum alanine and glutamine levels elevated, and serum phenylalanine levels reduced. Moreover, serum choline levels elevated (Additional file 1: Table S2).

Figures 2B, C, 3B, C show the results of the multivariate analysis of serum and urine samples based on pattern recognition on OPLS-DA. In both serum and urine samples, the groups differed in score plots. In addition, no overfitting risk was identified by permutation tests, suggesting successful establishment of the OPLS-DA model. A criterion combining multiple data processing methods, including FDR < 0.05 and VIP > 3 in serum and > 1 in urine, was used to select potential biomarkers with good accuracy. Compared with CAP without ARDS, we found that a series of metabolic changes in CAP with ARDS group, including higher levels of serum 3-hydroxybutyrate, lactate, acetone, and acetoacetate, and lower levels of serum leucine, choline and urine creatine, creatinine (Figs. 2B, 3B). Similarly, metabolic patterns changed after treatment in the CAP with ARDS group. The decreased serum 3-hydroxybutyrate, acetone, acetoacetate and elevated serum citrate, choline and urine 1-methylnicotinamide contributed to the difference after treatment in the CAP with ARDS group (Figs. 2C, 3C).

### AUC of significant metabolites in serum and urine for assessing therapeutic effects

**Fig. 4:**
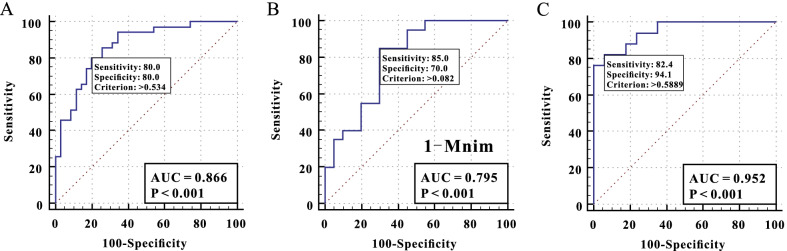
Receiver operating characteristic (ROC) curves and areas under the ROC curve (AUCs) before and after treatment in the community-acquired pneumonia with acquired respiratory distress syndrome group. **A** Combined ROC curve plot based on metabolites screened by VIP selected from serum metabolites. **B** ROC curve and AUC of the 1-methylnicotinamide screened based on VIP selected from urine metabolites. **C** ROC curve and AUC based on a total of associated serum and urine metabolites screened by the selected VIP. *3-HB* 3-hydroxybutyrate, *Acn* acetone, *Aca* acetoacetate, *Cit* citrate, *Cho* choline, *1-Mnim* 1-methylnicotinamide

Furthermore, to assess therapeutic effects on CAP with ARDS, diagnostic capacity of all key metabolites in blood or urine were screened based on VIP. ROC curves of combined serum metabolites 3-hydroxybutyrate, acetone, acetoacetate, citrate, and choline showed an AUC of 0.866, *p* < 0.001, sensitivity of 80.0%, and specificity of 80.0% (Fig. 4A) and that of the urine metabolite 1-methylnicotinamide showed an AUC of 0.795, *p* < 0.001, sensitivity of 85.0%, and specificity of 70.0% (Fig. 4B).

Subsequently, we combined all key metabolites found in blood and urine to determine the diagnostic capacity. Metabolic panels based on the cluster of characteristic serum and urine metabolites successfully evaluated therapeutic effects on CAP with ARDS with an AUC of 0.952, *p* < 0.001, sensitivity of 82.4%, and specificity of 94.1% (Fig. 4C).

### Metabolic pathway analysis and correlation of metabolites with clinical parameters

**Fig. 5:**
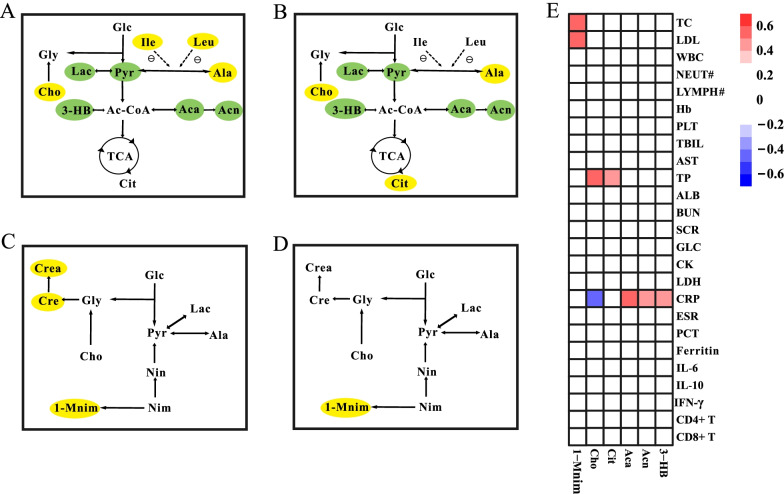
Metabolic pathway analysis. Metabolic pathway changes in serum between **A** community-acquired pneumonia (CAP) with and without acquired respiratory distress syndrome (ARDS) groups; and **B** before and after treatment in the CAP with ARDS group. Metabolic pathway changes in urine between **C** CAP with and without ARDS groups; and **D** before and after treatment in the CAP with ARDS group. The yellow or green shades represent significantly increased and decreased levels of metabolites, respectively. **E** Correlation heatmap of metabolites with clinical parameters in the community-acquired pneumonia with acute respiratory distress syndrome. Only correlation coefficients > 0.4 (moderate correlation) and *p*-values > 0.05 are highlighted. Red and blue colors indicate positive and negative correlations, respectively, with darker colors indicating higher correlations. *Cho* choline, *Gly* glycine, *Glc* glucose, *Pyr* pyruvate, *Lac* lactate, *Ile* isoleucine, *Leu* leucine, *Ala* alanine, *3-HB* 3-hydroxybutyrate, *CoA* coenzyme A, *Aca* acetoacetate, *Acn* acetone, *TCA* tricarboxylic acid, *Cit* citrate, *Cre* creatine, *Crea* creatinine, *Nin* nicotinate; *Nim* nicotinamide, *1-Mnim* 1-methylnicotinamide; *TC* total cholesterol, *LDL* low-density lipoprotein, *WBC* white blood cell; NEUT#, absolute neutrophil count; LYMPH#, absolute lymphocyte count, *Hb* hemoglobin, *PLT* platelet, *TBIL* total bilirubin, *AST* aspartate aminotransferase, *TP* total protein, *ALB* albumin, *BUN* blood urea nitrogen, *SCR* serum creatinine; GLC, glucose; *CK* creatine kinase, *LDH* lactate dehydrogenase; *CRP* C-reactive protein, *ESR* erythrocyte sedimentation rate, *PCT* procalcitonin, *IL* interleukin, *IFN* interferon, *CD4* cluster of differentiation 4; *CD8* cluster of differentiation 8

Significant metabolites changed in serum (Fig. 5A, B) and urine (Fig. 5C, D) samples as shown by the metabolic pathway. In comparisons of CAP with and without ARDS and CAP with ARDS before and after treatment, the energy, amino acid, and lipid metabolisms in serum and energy metabolism in urine differed. Figure 5E shows the results of the correlation analysis of differential metabolites in serum and urine with clinical parameters in patients with CAP with ARDS. The serum citrate level positively correlated with the total protein level. The serum choline level positively and negatively correlated with total protein and CRP levels, respectively. The CRP level positively correlated with serum acetoacetate, acetone, and 3-hydroxybutyric acid levels. The urine 1-methylnicotinamide level positively correlated with total cholesterol and low-density lipoprotein levels.

## Discussion

Exploring metabolic characterization of patients in CAP with ARDS and understand the potential metabolic mechanism underlying the progression of CAP and provide potential therapeutic strategy is important. In the present study, 20 serum metabolome signals and 42 urine metabolome signals were identified using NMR spectroscopy. Metabolites differed significantly between CAP with and without ARDS and before and after treatment in CAP with ARDS. Simultaneously, different metabolites showed specific changes in the metabolic pathway and reflected different disease stages. Differential serum metabolites were mainly involved in energy, lipid, and amino acid metabolisms, while differential urine metabolites were mainly involved in energy metabolism.

Disturbance in energy metabolism is a common metabolic feature of the progression of pneumonia[12–14]. We found elevated serum lactate levels in the CAP with ARDS group, consistent with laboratory findings. Lactate is a product of anaerobic glycolysis[15]. Lactate production increases when demands for ATP and oxygen exceed supplies. In our study, elevated lactate levels might indicate anaerobic glycolysis of glucose in the CAP with ARDS group. Hu et al.[16] also reported elevated lactate levels in a model of silica-induced lung inflammation and indicated alterations in the cellular energy pathways. A recent study[17] proposed that lactate is not a metabolic waste product but can contribute to energy metabolism as a major circulating carbohydrate fuel.

Our study also showed reduced levels of host-derived metabolites, such as urine creatine and creatinine, in the CAP with ARDS group. Further, serum creatine levels were significantly elevated in patients with CAP with ARDS after treatment. Creatinine is a breakdown product of creatine[18] that is involved in energy supply, indicating requirements of ATP and fatty acids during the disease course. In addition to the metabolism, energetics, and antioxidant potential of creatine by which it scavenges and neutralizes reactive oxygen species, it is a potential adjuvant therapy for various diseases[19–21]. These data also suggest the clinical treatment utilities of creatine and creatinine in patients with CAP with ARDS.

As for energy metabolism, serum citrate levels were also elevated after treatment and positively correlated with total protein levels. Citrate is synthesized by citrate synthase and is an intermediate in the interconversion of various substances in the body[22]. It serves as a consuming substrate for the tricarboxylic acid (TCA) cycle, increasing production of ATP via the TCA cycle with oxidative phosphorylation pathways in ARDS. The upregulated TCA cycle plays a role in supporting bioenergetics under hypoxic and energy depletion conditions. In addition, conversion of citrate to acetyl coenzyme A is important for protein acetylation, which has been linked to macrophage and dendritic cell activations[23]. The citrate pathway likely plays a role in regulating immune cell metabolism and producing pro-inflammatory mediators[24, 25].

In our study, urine 1-methylnicotinamide levels were also elevated after treatment and positively correlated with total cholesterol and low-density lipoprotein levels. 1-Methylnicotinamide is converted from nicotinamide, which is a form of vitamin B_3_ and precursor for nicotinamide adenine dinucleotide, and crucial in modulating energy metabolism and preventing oxidative stress[26, 27]. Nicotinamide has been studied as a novel therapeutic candidate in lung injury[28–30] and was found to significantly improve lipid metabolism in humans[31, 32].

Furthermore, the analysis of serum metabolites revealed the presence of general metabolic stress in patients with CAP with ARDS, as indicated by elevated serum acetoacetate, acetone, and 3-hydroxybutyric acid levels in the CAP with ARDS group compared to the CAP without ARDS group. Moreover, the levels of all the three serum metabolites decreased after treatment in the CAP with ARDS group, and CRP levels positively correlated with serum acetoacetate, acetone, and 3-hydroxybutyric acid levels. Ketone bodies (including acetoacetate, acetone, and 3-hydroxybutyric acid) are produced predominantly in the liver from fatty acid oxidation-derived acetyl coenzyme A and transported to the extrahepatic tissues for oxidation. The presence of ketone bodies indicates fatty acid oxidation activation due to energy metabolism and ATP-deficit disorders[33]. As a terminal product of fatty acid β-oxidation, increased 3-hydroxybutyrate typically reflects fat utilization as an energy source to compensate for the increased ketone body energetic demands following tissue damage[34]. Breath acetone is a marker of ischemia–reperfusion-induced oxidative stress, which causes lipid peroxidation and plays a key role in the early stage of ARDS[35]. A recent study showed that acetoacetate, 3-hydroxybutyric acid, and acetone were markedly elevated in the serum of patients with coronavirus disease[36]. In a previous study, 3-hydroxybutyric acid levels were elevated in complicated parapneumonic effusion and could lead to the requirement of aggressive pleural drainage[37]. These conclusions highlight the increased energy requirement during lung injury, and the aforementioned metabolite changes could provide energy and nutrients for the process of lung injury[38].

As for amino acid metabolism, the present study showed lower levels of leucine and isoleucine in the serum of patients with CAP with ARDS than in those with CAP without ARDS. Similarly, branched-chain amino acids (BCAAs) were lowered below normal levels in brain injury[39, 40]. BCAAs include valine, leucine, and isoleucine. The reason and significance of BCAA reduction in patients with ARDS are unclear. However, immune cells oxidize BCAAs as fuel sources and incorporate them as vital components of the immune-enhancing formula[41]. In recent years, there has been growing interest in the role of BCAAs in immune function. Nakmura[42] proved that oral supplementation of BCAAs improved phagocytic function of neutrophils and natural killer cell activity of lymphocytes in patients with liver cirrhosis. Thus, supplementing BCAAs might improve immune function, which could be conducive to the recovery of ARDS.

In lipid metabolism, serum choline levels were lower in the CAP with ARDS group than in the CAP without ARDS group and significantly increased after treatment in the ARDS group. Choline is a precursor of phosphatidylcholine biosynthesis, the main component of cellular membranes[43]. Membrane phosphatidylcholine can be utilized to generate pro- and anti-inflammatory lipid mediators, which can contribute to the pathogenesis of ARDS[44]. Choline uptake is essential for phospholipid remodeling and maintaining mitochondrial function and integrity in metabolically challenged macrophages to make up for the energy cost of inflammatory cytokine production and bactericidal activity and maintaining membrane stability[43]. In addition, the choline level positively and negatively correlated with total protein and CRP levels in the CAP with ARDS group. Systemic inflammation affecting the liver can produce acute-phase proteins[45]. Choline therapy modulates immune inflammation and suppresses oxidative stress, eliciting various pharmacological effects in many diseases[46–49]. Our data also offer promising choline therapeutic strategies for ARDS.

The present study demonstrated that a wide range of metabolites associated with cellular pathways can be accurately analyzed. The ROC analysis of related differential metabolites revealed that the diagnostic efficacy of serum metabolites was better than that of urine metabolites. Combined serum and urine metabolites had the highest AUC for evaluating therapeutic effects on CAP with ARDS (0.952; sensitivity, 82.4%; specificity, 94.1%). However, this study had some limitations. First, the sample size was small. Second, the detected significant metabolites were specific to CAP-induced ARDS was not determined. Third, only Chinese patients were enrolled, and diagnostic criteria of CAP in China were applied. Therefore, further large-scale studies with greater generalizability should be performed to verify the accuracy of serum and urine metabolites as novel markers for metabolic characterization of CAP with ARDS and prediction of treatment success.

## Conclusions

In this study, we investigated serial serum and urine metabolomic profiles in patients with CAP with and without ARDS using NMR spectroscopy and presented the complex biological process and interconnected metabolism by conducting a pathway analysis and analyzing the relationship between alterations in metabolites and clinical features. Changes in the identified significant metabolites, indicating derangements of energy, lipid, and amino acid metabolisms, may shed light on the mechanisms involved in the pathogenesis of CAP with ARDS and provide new drug targets for the clinical prevention and treatment. Our data also suggest characteristic metabolic alterations as evaluation of therapeutic effect indicators of CAP with ARDS.

## Supplementary Information

**Additional file 1: Table S1.** Clinical characteristics before and after treatment of patients with community-acquired pneumonia with acute respiratory distress syndrome. **Table S2.** Metabolite changes in serum and urine

## Data Availability

The datasets used and/or analyzed during the current study can be obtained from the corresponding author on reasonable request.

## Abbreviations

ARDS: Acute respiratory distress syndrome
CAP: Community-acquired pneumonia
ROC: Receiver operating characteristic
NMR: Nuclear magnetic resonance
AUC: Area under the receiver operating characteristic curve
TSP: 3-(Trimethylsilyl) propionic-2,2,3,3,d4
OPLS-DA: Orthogonal partial least-squares discriminant analysis
VIP: Variable importance in projection
FDR: False discovery rate
WBC: White blood cell
CRP: C-reactive protein
TCA: Tricarboxylic acid
BCAA: Branched-chain amino acid
