# Supplementary material for: Metabolomics profile in acute respiratory distress syndrome by nuclear magnetic resonance spectroscopy in patients with community-acquired pneumonia
Source: Respir Res. 2022 Jun 27;23:172. doi: 10.1186/s12931-022-02075-w (PMC9235271; doi:10.1186/s12931-022-02075-w)
Supplement: Supplementary file 1 — Additional file 1: Table S1. Clinical characteristics before and after treatment of patients with community-acquired pneumonia with acute respiratory distress syndrome. Table S2. Metabolite changes in serum and urine [file 12931_2022_2075_MOESM1_ESM.docx]

**Table S1.** Clinical characteristics before and after treatment of patients with community-acquired pneumonia with acute respiratory distress syndrome

| **Variables** | **CAP with ARDS (n = 43)** | | | | | |
| --- | --- | --- | --- | --- | --- | --- |
|  | **Before** | **After** | ***P*** | **FDR** | **95% Cl** | |
|  |  |  |  |  | **Lower** | **Higher** |
| WBC (×10^9/L) | 10.2 (5.9–13.1) | 6.4 (4.3–8.4) | 0.001 | 0.001 | 7.3 | 9.7 |
| NEUT# (×10^9/L) | 8.7 (5.0–11.4) | 4.5 (2.9–6.1) | 0.000 | 0.000 | 5.6 | 8.0 |
| LYMPH# (×10^9/L) | 0.8 (0.4–1.2) | 1.1 (0.7–1.5) | 0.001 | 0.001 | 1.0 | 1.2 |
| Hb (g/L) | 113 (95–122) | 103 (86–116) | 0.000 | 0.000 | 99.0 | 106.2 |
| PLT (×10^9/L) | 164 (88–237) | 242 (172–357) | 0.001 | 0.001 | 211.4 | 260.7 |
| ALT (U/L） | 34 (25–58) | 36 (22–53) | 0.319 | 0.363 | 25.1 | 87.4 |
| AST (U/L） | 51 (32–74) | 33 (23–56) | 0.005 | 0.008 | 7.2 | 221.0 |
| TBIL (µmol/L) | 13 (8–24) | 11 (8–14) | 0.029 | 0.038 | 13.4 | 20.1 |
| TP (g/L) | 53.2 ± 6.8 | 62.9±8.5 | 0.000 | 0.000 | 58.0 | 61.2 |
| ALB (g/L) | 26.3 ± 4.0 | 31.7±4.7 | 0.000 | 0.000 | 29.0 | 30.8 |
| BUN (mmol/L) | 6.8 (4.6–10.4) | 4.6 (3.6–8.1) | 0.010 | 0.014 | 6.3 | 9.0 |
| SCR (µmol/L) | 74 (50–117) | 64 (53–80) | 0.002 | 0.003 | 71.8 | 94.1 |
| BUA (µmol/L) | 231.8 ± 115.6 | 191.7±81.4 | 0.051 | 0.065 | 188.5 | 221.7 |
| GLC (µmol/L) | 7.6 (5.8–9.9) | 6.2 (5.0–9.3) | 0.013 | 0.018 | 7.4 | 9.0 |
| CK (µ/L) | 64 (30–222) | 32 (22–50) | 0.000 | 0.000 | 14.0 | 377.7 |
| LDH (µ/L) | 377 (251–502) | 262 (198–337) | 0.000 | 0.000 | 260.9 | 614.0 |
| IL-2 (µg/L) | 0.4 (0.1–0.6) | 0.5 (0.1–0.8) | 0.355 | 0.379 | 0.4 | 0.6 |
| IL-4 (µg/L) | 0.1 (0.1–0.7) | 0.3 (0.1–0.6) | 1.000 | 1.000 | 0.3 | 1.3 |
| IL-6 (µg/L) | 208.3 (97.4–1783.9) | 13.5 (7.8–37.0) | 0.000 | 0.000 | 681.0 | 2065.8 |
| IL-10 (µg/L) | 8.4 (5.3–36.4) | 4.7 (2.8–6.3) | 0.000 | 0.000 | 15.1 | 61.5 |
| TNF-α (pg/mL) | 1.0 (0.6–2.6) | 1.0 (0.5–10.6) | 0.388 | 0.400 | 4.3 | 9.5 |
| IFN-γ (µg/L) | 1.2 (0.2–3.5) | 0.8 (0.2–1.6) | 0.002 | 0.003 | 2.4 | 9.0 |
| CD4+ T (/UI） | 254 (149–382) | 464.0 (243.0–614.0) | 0.000 | 0.000 | 353.2 | 450.4 |
| CD8+T (/UI） | 193 (90–272) | 312.7 (180.0–424.0) | 0.000 | 0.000 | 251.6 | 315.7 |
| D–D (mg/L) | 2.9 (2.0–6.2) | 3.0 (1.5–4.9) | 0.128 | 0.156 | 3.8 | 5.2 |
| CRP (mg/dL) | 183 (146–215) | 23.1 (12.8–66.2) | 0.000 | 0.000 | 82.0 | 119.5 |
| PCT (ng/mL) | 5.7 (0.9–18.3) | 0.1 (0.1–0.4) | 0.000 | 0.000 | 4.0 | 12.3 |
| ESR (mm/h) | 42.0 (25.0–59.8) | 33.0 (17.9–56.0) | 0.007 | 0.010 | 35.4 | 43.5 |
| Ferritin (µg/L) | 1018.6 (475.5–1500.0) | 645.5 (350.0–1167.0) | 0.004 | 0.007 | 790.1 | 1360.6 |
| TC (mmol/L) | 3.0 ± 1.0 | 3.7 ± 1.2 | 0.000 | 0.000 | 3.2 | 3.6 |
| TG (mmol/L) | 1.6 (0.9–1.9) | 1.5 (1.2–2.0) | 0.356 | 0.379 | 1.5 | 2.0 |
| HDL (mmol/L) | 0.6 (0.3–0.9) | 0.6 (0.5–0.7) | 0.274 | 0.323 | 0.6 | 0.7 |
| LDL (mmol/L) | 1.5 ± 0.8 | 2.2 ± 0.9 | 0.000 | 0.000 | 1.7 | 2.1 |

Abbreviation: CAP, community-acquired pneumonia; ARDS, acute respiratory distress syndrome; WBC, white blood cell; NEUT#, absolute neutrophil count; LYMPH#, absolute value of lymphocytes; Hb, hemoglobin; PLT, platelets; ALT, alanine aminotransferase; AST, aspartate amino transferase; TBIL, total bilirubin; TP, total protein; ALB, albumen; BUN, blood urea nitrogen; SCR, serum creatinine; GLC, glucose; CK, creatine kinase; LDH, lactate dehydrogenase; IL, interleukin; TNF, tumor necrosis factor; IFN, interferon; CD4, cluster of differentiation 4; CD8, cluster of differentiation 8; D-D, d dimer; CRP, c-reactive protein; PCT, procalcitonin; ESR, erythrocyte sedimentation rate; TC, total cholesterol; TG, triglycerides; HDL, high density lipoprotein; LDL, low-density lipoprotein.

**Table S2.** Metabolite changes in serum and urine

| **Serum** | **CAP with/without ARDS** | | | | | |  | **Before-/After- treatment in ARDS** | | | | | |
| --- | --- | --- | --- | --- | --- | --- | --- | --- | --- | --- | --- | --- | --- |
| **Metabolite（r.u.）** | **CAP with ARDS** | **CAP without ARDS** | ***P*** | **FDR** | **95%Cl** | |  | **Before** | **After** | ***P*** | **FDR** | **95%Cl** | |
|  |  |  |  |  | **Lower** | **Higher** |  |  |  |  |  | **Lower** | **Higher** |
| Methylhistidine | 0.32±0.11 | 0.34±0.08 | 0.293 | 0.879 | 0.31 | 0.35 |  | 0.32±0.08 | 0.31±0.06 | 0.750 | 0.891 | 0.30 | 0.33 |
| Phenylalanine | 2.41±0.87 | 1.93±0.5 | 0.002 | 0.006 | 2.00 | 2.33 |  | 2.4±0.82 | 1.75±0.6 | 0.000 | 0.000 | 1.88 | 2.25 |
| Tyrosine | 0.67±0.26 | 0.75±0.16 | 0.085 | 0.255 | 0.66 | 0.76 |  | 0.67±0.27 | 0.71±0.24 | 0.335 | 0.530 | 0.63 | 0.76 |
| Lactate | 7.61±3.38 | 6.11±1.89 | 0.010 | 0.030 | 6.24 | 7.47 |  | 7.82±3.52 | 6.28±2.27 | 0.001 | 0.003 | 6.32 | 7.74 |
| Glucose | 486.9±77.17 | 497.55±60.07 | 0.472 | 1.000 | 477.26 | 507.32 |  | 487.87±77.84 | 479.38±62.61 | 0.559 | 0.801 | 464.93 | 498.37 |
| Choline | 58.28±11.57 | 65.99±11.99 | 0.008 | 0.023 | 59.48 | 64.87 |  | 57.85±11.15 | 70.97±15.42 | 0.000 | 0.000 | 61.04 | 67.95 |
| Creatine | 3.19±1.24 | 2.7±0.61 | 0.015 | 0.045 | 2.72 | 3.16 |  | 3.25±1.22 | 3.31±0.69 | 0.667 | 0.845 | 3.08 | 3.55 |
| Citrate | 43.33±8.01 | 48.14±9.41 | 0.034 | 0.103 | 43.80 | 47.74 |  | 43.21±7.7 | 51.9±13.06 | 0.000 | 0.000 | 44.92 | 50.25 |
| Glutamine | 4.14±1.36 | 4.81±1.08 | 0.014 | 0.043 | 4.20 | 4.75 |  | 4.11±1.25 | 5.54±1.24 | 0.000 | 0.000 | 4.50 | 5.17 |
| Pyruvate | 4.67±2.22 | 3.71±0.9 | 0.005 | 0.015 | 3.81 | 4.57 |  | 4.78±2.34 | 3.82±1.07 | 0.018 | 0.034 | 3.83 | 4.70 |
| Acetoacetate | 7.88±5.66 | 5.49±2.15 | 0.005 | 0.014 | 5.71 | 7.63 |  | 8.15±5.96 | 4.7±2.17 | 0.002 | 0.005 | 5.25 | 7.48 |
| Acetone | 8.33±3.76 | 6.55±1.85 | 0.003 | 0.009 | 6.76 | 8.10 |  | 8.49±3.99 | 6.24±1.92 | 0.003 | 0.007 | 6.62 | 8.17 |
| Acetate | 48.32±9.27 | 50.11±7.59 | 0.330 | 0.990 | 47.38 | 51.07 |  | 47.91±9.31 | 49.61±8 | 0.280 | 0.484 | 46.87 | 50.94 |
| Alanine | 4.64±1.52 | 5.82±2.22 | 0.004 | 0.011 | 4.80 | 5.67 |  | 4.69±1.55 | 5.98±1.52 | 0.000 | 0.000 | 4.99 | 5.78 |
| 3-hydroxybutyric acid | 13.41±6.97 | 10.08±2.89 | 0.003 | 0.008 | 10.52 | 12.94 |  | 13.62±7.37 | 10.08±3.87 | 0.010 | 0.021 | 10.35 | 13.19 |
| Valine | 3.18±0.95 | 3.53±0.78 | 0.057 | 0.172 | 3.17 | 3.55 |  | 3.25±0.91 | 3.26±0.74 | 0.948 | 0.980 | 3.08 | 3.52 |
| Isoleucine | 4.83±1.31 | 5.54±0.96 | 0.004 | 0.013 | 4.93 | 5.45 |  | 4.92±1.26 | 4.9±1.06 | 0.935 | 0.980 | 4.67 | 5.29 |
| Leucine | 13.25±2.96 | 14.79±1.9 | 0.004 | 0.012 | 13.46 | 14.59 |  | 13.36±2.77 | 13.08±2.1 | 0.590 | 0.801 | 12.74 | 14.10 |
| LDL/VLDL | 54.03±16.25 | 52.66±13.47 | 0.669 | 1.000 | 50.10 | 56.58 |  | 53.48±15.08 | 53.54±13.6 | 0.980 | 0.980 | 50.42 | 57.20 |
|  |  |  |  |  |  |  |  |  |  |  |  |  |  |
|  |  |  |  |  |  |  |  |  |  |  |  |  |  |
| **Urine** | **CAP with/without ARDS** | | | | | |  | **Before-/After- treatment in ARDS** | | | | | |
| **Metabolite（r.u.）** | **CAP with ARDS** | **CAP without ARDS** | ***P*** | **FDR** | **95%Cl** | |  | **Before** | **After** | ***P*** | **FDR** | **95%Cl** | |
|  |  |  |  |  | **Lower** | **Higher** |  |  |  |  |  | **Lower** | **Higher** |
| 1-Methylnicotinamide | 0.08±0.03 | 0.12±0.09 | 0.015 | 0.045 | 0.08 | 0.12 |  | 0.08±0.03 | 0.12±0.03 | 0.000 | 0.000 | 0.09 | 0.11 |
| Trigonelline | 0.27±0.19 | 0.28±0.17 | 0.876 | 1.000 | 0.23 | 0.32 |  | 0.29±0.21 | 0.29±0.11 | 0.918 | 0.944 | 0.24 | 0.34 |
| Formate | 0.94±0.5 | 0.86±0.42 | 0.540 | 1.000 | 0.78 | 1.01 |  | 0.93±0.45 | 1.14±0.5 | 0.096 | 0.273 | 0.88 | 1.19 |
| Hippurate | 1.03±0.6 | 0.73±0.52 | 0.096 | 0.289 | 0.71 | 1.01 |  | 0.39±0.43 | 0.66±0.67 | 0.724 | 0.873 | 0.88 | 1.39 |
| Histidine | 0.44±0.42 | 0.5±0.61 | 0.681 | 1.000 | 0.34 | 0.61 |  | 0.44±0.42 | 0.55±0.55 | 0.051 | 0.226 | 0.34 | 0.71 |
| Tyrosine | 1.72±1.08 | 2.16±1.16 | 0.137 | 0.412 | 1.68 | 2.27 |  | 1.63±0.96 | 1.7±0.99 | 0.793 | 0.873 | 1.36 | 1.97 |
| N-Methylhistidine | 0.67±0.62 | 0.51±0.51 | 0.247 | 0.740 | 0.44 | 0.73 |  | 0.57±0.52 | 0.47±0.39 | 0.510 | 0.786 | 0.37 | 0.67 |
| Vanillate | 1.02±0.46 | 0.98±0.43 | 0.697 | 1.000 | 0.88 | 1.11 |  | 1.01±0.4 | 1.14±0.38 | 0.154 | 0.380 | 0.95 | 1.20 |
| Fumarate | 0.09±0.08 | 0.09±0.08 | 0.990 | 1.000 | 0.07 | 0.11 |  | 0.09±0.09 | 0.17±0.16 | 0.021 | 0.226 | 0.09 | 0.18 |
| Urea | 163.92±74.41 | 183.71±104.29 | 0.415 | 1.000 | 151.19 | 199.46 |  | 169.05±79 | 214.43±88.1 | 0.085 | 0.273 | 164.33 | 219.16 |
| Glucose | 7.64±11.33 | 8.07±9.96 | 0.872 | 1.000 | 2.34 | 3.50 |  | 6.99±10.04 | 8.4±8.05 | 0.628 | 0.873 | 4.82 | 10.58 |
| Creatinine | 27.54±9.83 | 38.35±14.39 | 0.002 | 0.005 | 30.21 | 37.33 |  | 27.88±9.63 | 33.98±12.12 | 0.032 | 0.226 | 27.33 | 34.52 |
| Glycylproline | 6.5±5.64 | 4.28±1.75 | 0.021 | 0.062 | 4.17 | 6.27 |  | 6.09±5.48 | 5.03±2.37 | 0.303 | 0.534 | 4.22 | 6.90 |
| Choline | 6.8±4.32 | 11.79±12.74 | 0.051 | 0.154 | 6.99 | 12.36 |  | 6.52±3.64 | 10.15±7.86 | 0.035 | 0.226 | 6.32 | 10.36 |
| Betaine | 3.66±4.94 | 4.61±4.22 | 0.364 | 1.000 | 3.03 | 5.38 |  | 3.34±4.58 | 2.37±0.97 | 0.351 | 0.590 | 1.80 | 3.91 |
| N，N-Dimethylglycine | 1.09±0.52 | 1.16±0.44 | 0.575 | 1.000 | 1.01 | 1.26 |  | 1.05±0.51 | 1.09±0.47 | 0.762 | 0.873 | 0.92 | 1.23 |
| Dimethylamine | 2.89±0.96 | 3.58±1.7 | 0.072 | 0.217 | 2.90 | 3.67 |  | 2.79±0.87 | 3.47±1.39 | 0.054 | 0.226 | 2.75 | 3.51 |
| Citrate | 2.39±1.42 | 2.64±1.48 | 0.585 | 1.000 | 2.15 | 2.91 |  | 2.2±1.17 | 3.76±2.36 | 0.008 | 0.148 | 2.34 | 3.62 |
| Acetate | 1.87±1.02 | 1.93±0.95 | 0.829 | 1.000 | 1.65 | 2.16 |  | 1.72±0.87 | 2.2±1.45 | 0.259 | 0.532 | 1.58 | 2.35 |
| Alanine | 1.86±1.23 | 1.55±1.02 | 0.386 | 1.000 | 1.39 | 1.97 |  | 1.88±1.26 | 1.98±1.89 | 0.813 | 0.873 | 1.43 | 2.44 |
| 2-Hydroxyisobutyrate | 1.14±0.53 | 0.99±0.43 | 0.208 | 0.625 | 0.93 | 1.18 |  | 1.22±0.54 | 1.06±0.43 | 0.285 | 0.534 | 0.98 | 1.29 |
| Lactate | 3.69±1.54 | 3.86±2.49 | 0.756 | 1.000 | 3.24 | 4.34 |  | 3.94±1.46 | 4.1±1.44 | 0.729 | 0.873 | 3.56 | 4.48 |
| Hypoxanthine | 0.73±0.26 | 0.71±0.32 | 0.713 | 1.000 | 0.64 | 0.79 |  | 0.74±0.26 | 0.76±0.17 | 0.747 | 0.873 | 0.68 | 0.82 |
| Theophylline | 0.74±0.41 | 0.85±0.76 | 0.476 | 1.000 | 0.64 | 0.97 |  | 0.77±0.43 | 0.74±0.39 | 0.787 | 0.873 | 0.62 | 0.88 |
| 3-Indoxylsulface | 0.82±0.35 | 0.9±0.68 | 0.562 | 1.000 | 0.72 | 1.01 |  | 0.85±0.35 | 0.92±0.42 | 0.430 | 0.692 | 0.76 | 1.01 |
| Tartrate | 0.41±0.38 | 0.52±0.79 | 0.601 | 1.000 | 0.30 | 0.64 |  | 0.36±0.34 | 0.77±1.1 | 0.065 | 0.241 | 0.30 | 0.83 |
| Fructose | 5.16±1.47 | 4.83±1.62 | 0.426 | 1.000 | 4.56 | 5.37 |  | 4.96±1.32 | 5.95±1.65 | 0.042 | 0.226 | 4.96 | 5.95 |
| Gluconate | 2.75±1.7 | 2.69±1.24 | 0.866 | 1.000 | 2.34 | 3.09 |  | 2.55±1.52 | 2.74±1.16 | 0.655 | 0.873 | 2.22 | 3.07 |
| Threonine | 10.45±5.74 | 9.03±3.73 | 0.220 | 0.661 | 8.41 | 10.85 |  | 10.51±6.11 | 8.48±3.24 | 0.206 | 0.448 | 7.91 | 11.07 |
| Glycine | 4.67±1.87 | 4.74±1.86 | 0.900 | 1.000 | 4.23 | 5.19 |  | 4.75±1.71 | 5.59±2.19 | 0.089 | 0.273 | 4.53 | 5.81 |
| Creatine | 40.38±17.3 | 55.23±25.82 | 0.013 | 0.040 | 42.78 | 55.09 |  | 39.57±17.71 | 46.36±21.08 | 0.191 | 0.442 | 36.72 | 49.21 |
| Asparagine | 1.18±0.59 | 1.25±0.75 | 0.704 | 1.000 | 1.04 | 1.39 |  | 1.18±0.62 | 1.17±0.65 | 0.981 | 0.981 | 0.97 | 1.37 |
| Acetone | 1.63±1.22 | 1.39±1.56 | 0.644 | 1.000 | 1.12 | 1.86 |  | 1.5±1.23 | 1.67±2.99 | 0.826 | 0.873 | 0.86 | 2.31 |
| 3-Hydroxyisovalerate | 3.11±3.02 | 1.89±1.28 | 0.027 | 0.081 | 1.82 | 2.99 |  | 2.43±1.8 | 2.07±1.55 | 0.299 | 0.534 | 1.72 | 2.78 |
| Tryptamine | 1.74±1.2 | 1.73±1.89 | 0.977 | 1.000 | 1.31 | 2.15 |  | 1.75±1.28 | 1.09±0.86 | 0.055 | 0.226 | 1.06 | 1.78 |
| Taurine | 16.87±24.75 | 17.13±21.13 | 0.960 | 1.000 | 11.15 | 22.89 |  | 14.74±21.79 | 7.39±3.39 | 0.118 | 0.312 | 6.00 | 16.13 |
| Acetamide | 1.15±0.46 | 1.19±0.56 | 0.813 | 1.000 | 1.04 | 1.31 |  | 1.13±0.45 | 1.19±0.44 | 0.611 | 0.873 | 1.02 | 1.30 |

Abbreviation: CAP, community-acquired pneumonia; ARDS, acute respiratory distress syndrome; LDL/VLDL, low-density lipoprotein/very-low-density lipoprotein
